# The prevalence of foot health problems in people living with a rheumatic condition: a cross-sectional observational epidemiological study

**DOI:** 10.1007/s00296-022-05236-8

**Published:** 2022-10-20

**Authors:** Minna Stolt, Anne-Marie Laitinen, Katja Kankaanpää, Jouko Katajisto, Lindsey Cherry

**Affiliations:** 1 Department of Nursing Science, University of Turku, 20014 Turku, Finland; 2 Department of Nursing Science, University of Eastern Finland, Kuopio, Finland; 3 Department of Mathematics and Statistics, University of Turku, Turku, Finland; 4 School of Health Sciences, University of Southampton, Southampton, UK

**Keywords:** Foot health, Foot pain, Survey, Epidemiology, Rheumatic conditions, Self-care

## Abstract

This study aimed to determine the prevalence of foot health problems in people living with any rheumatic condition and explore potential associations with exposure variables. A cross-sectional observational epidemiological design was applied. The participants were recruited from one regional patient association in southwest Finland. The data were collected in January–February 2019 and included the Self-reported Foot Health Assessment Instrument (S-FHAI) and demographic questions. In total, 495 responses were obtained. Overall, participants had many foot problems. The point prevalence of self-reported foot problems was 99 per 100 people living with a rheumatic condition. The most prevalent problems were foot pain (73%), dry soles (68%), thickened toenails (58%) and cold feet (57%). Lower educational attainment, increased amount of daily standing and accessing medical or nursing care for foot problems were associated with poorer foot health. The results reveal a high frequency of foot pain among people with rheumatic conditions. The study highlighted the importance of person-centred care and the biological focus that underpins and impacts foot health (what we understand, what we do, and our health-seeking behaviour). Interventions to promote biopsychosocial approaches to personalised foot care could advance people’s readiness, knowledge and skill to care for their own feet.

## Introduction

Foot health in people with rheumatic conditions is constantly changing due to the auto-immune mediated inflammatory and progressive nature of such disease [1–3], of which RA and OA are the most common [4]. Rheumatic conditions are prevalent long-term health conditions, and it is estimated that the worldwide prevalence proportion of long-term inflammatory rheumatic disease is approximately 5% [5]. Typical symptoms in rheumatic conditions include joint inflammation driving pain, stiffness and/or swelling, particularly in the foot and ankle [6]. Persistent inflammation leads to progressive non-reversible joint damage, resulting in increasing levels of disability and decreased quality of life [7]. Foot problems in people living with rheumatic conditions cause significant restrictions to their activities of daily living and quality of life [8–10].

Previous foot health research among people with rheumatic conditions has focused specifically on foot health in focal patient groups, such as people living with rheumatoid arthritis, psoriatic arthritis, osteoarthritis or systemic lupus erythematosus [11–14]. Common to all these rheumatic conditions is a high frequency of foot problems [15, 16], many of which statistically demonstrate bio-psycho-socio-demographic association in cross-sectional analyses [11, 17, 18]. Foot pain and foot structural deformities, such as toe deformities, and forefoot disorders, are the most common forms of foot problems in people with RA [15, 19], causing significant restrictions to quality of life [8]. For some people, foot problems are reported as impacting physical, social, and emotional lives [9].

However, whilst there is a range of cohort data available for some rheumatic diseases, most previous research has predominantly focused on reporting foot health status based on clinical, objective foot health assessment alone [15]; peoples’ self-reported assessment of their foot health is seldom studied. Moreover, in these previous studies, most participants have been recruited from a group of people attending primary or specialised care clinics because of their rheumatic disease or their foot problems. The studies may be disregarding people who do not need regular professional care for their rheumatic condition due to a systematic recruitment bias. Investigating a person’s view of their foot health and recruiting them outside of healthcare organisations could deepen the evidence on foot health among people with rheumatic conditions.

Healthcare utilisation by people living with complex rheumatic conditions is typically higher than a population average usage [20]. Previous researchers have recommended foot health assessment integral within episodes of healthcare contact [21], arguably identifying potential problems early and sign-posting toward or offering personalised therapeutic intervention if needed. However, as noted earlier, not all people living with rheumatic disease will routinely come into contact with specialist services and may be accessing support for foot health and care outside of this framework. Where national services or clinical guidelines are in place, the delivery and organisation of care still remains highly variable, and is likely to affect service utilisation, and therefore, any cohort data derived from clinical epidemiological study recruiting only via healthcare settings.

Many clinical practice guidelines relate to the management of rheumatic conditions exist, however, most concern pharmacological management [22], and there is minimal guidance relating to foot health. To the best of our knowledge, there are two national guidelines emphasising foot care; The United Kingdom's national guideline for RA advocates for an annual foot health review. However, the provision of dedicated foot care services or staff training to achieve this is variable [9]. In the Netherlands, multidisciplinary recommendations for managing foot problems in people with RA have been developed [23]; however, service structures to deliver this are fragmented. Care and training fragmentation was also identified in a pan-European, identifying only three countries, the Netherlands, the UK and Malta, has having any semi-specialised provision [24]. All in all, there is a lack of focused podiatric clinical care and guidelines for caring for patients with rheumatic condition-related foot and ankle problems.

Thus, this study aimed to investigate self-reported foot health in adults individuals diagnosed with rheumatic disease. To inform the adequate provision of services, delivered in a timely way that best meets the needs of intended end users, there is a need to determine an estimate of the point prevalence of foot problems within the population of people living with rheumatic conditions.

## Aim

The main aim of this study was to determine the prevalence of foot health problems in people living with any rheumatic condition. A secondary aim was to explore potential associations between level of foot health and pragmatically derived bio-psycho-social exposure variables. A series of research objectives were proposed:

1. To determine the self-reported point prevalence of foot problems in a cohort of people living with a rheumatic condition
2. To describe the range and frequency of foot problems reported
3. To explore the statistical association between the presence of foot problems and pragmatically derived bio-psycho-social exposure variables

## Methods

### Design

A cross-sectional observational epidemiological design was used to determine the point prevalence of foot problems within a sampling frame of all people living with a rheumatic condition within a single health region in Finland.

### Sampling

Study participants were recruited from one purposively selected regional patient association in southwest Finland. The patient associations work in the third-sector environment providing peer support, functional group exercises and leisure activities to people living with rheumatic conditions. The paper-based survey was sent by mail to all recorded members of the selected patient association, which determined the pragmatic sampling frame and size for this study (*n* = 1318). A single electronic reminder to complete the survey was sent to group members via their social media page and posted on the website 14 days after the initial survey distribution.

Previous estimates of foot problems within rheumatic populations have varied but remain relatively high at 20 + %. Assuming a lower expected frequency estimate of 10%, to account for a possible reduction in foot problems in groups as yet unaccounted for, a sample size of 864 is required to give a precise estimate within 2% [25]. Therefore, this pragmatic sampling frame was considered adequate to achieve the primary research objective.

The inclusion criteria for participation were: (1) consultant confirmed diagnosis of a rheumatic condition, (2) aged 18 years or over, and (3) able and willing to consent and complete the survey.

### Data collection

Data were collected in January–February 2019 using the Self-reported Foot Health Assessment Instrument (S-FHAI) [26]. The S-FHAI measures current levels of foot health as assessed by the person completing the form. It consists of 22 items divided into four subscales: skin (12 items), nails (4 items), foot structure (5 items) and foot pain (1 item) [26]. The response scale for each item is dichotomous (yes/no) [26]. The S-FHAI provides a total score ranging from 22 to 44 [26]. Higher scores indicate poorer foot health. The S-FHAI has previously been used in assessing nurses’ foot health, and its validity has been determined to be satisfactory [26]. For foot pain, questions about the location (toes, sole of the foot, heel, ankle, knee, thigh, hip) and incidence (4-point scale: slight, moderate, strong, worst imaginable) were also asked [26].

In addition, participants’ age (years), gender at birth (male/female), basic educational level (3-point scale: elementary, primary or high school*)*, professional education (5-point scale from no professional education to university degree), current occupation (6-point scale: manager, employee, self-employed, student, pensioner, unemployed), type of rheumatic condition, primarily used footwear style (walking shoes, athletics shoes, court shoes, protection shoes, barefoot shoes, sandals), primarily used type of sock (cotton socks, mixed material socks, compression socks, stockings) were recorded. In addition, questions relating to foot health and daily physical activity were asked, including perceived importance of foot health (5-point scale from very important to not very important), use of medical care for foot problems (yes–no), perceived impact of foot health on daily activities (5-point scale from very much to very little), self-assessed foot health score (0 = poorest foot health, 10 = best possible foot health), work absenteeism because of foot problems (yes–no), and amount of walking or standing per day (5-point scale from very much to very little). A pilot study was conducted with ten participants to analyse the clarity of the response instructions and items. No changes were made after the pilot study.

### Ethical considerations

The study followed good scientific practice at every stage [27]. Ethical approval from the University Ethical Review Board was obtained (Ethical Committee Code: 8/2018, date: 29.1.2018). Permission to conduct the data collection was approved by the executive committee of the patient association. Each eligible participant received a written cover letter about the study, stating the study’s purpose, voluntary participation, confidential data handling, anonymity of the responses, reporting the results and the right to withdraw at any stage. The eligible participants were provided with instructions on how to respond to the survey and the researcher’s contact details if they wanted to discuss or clarify any potentially unclear issues. Return of the completed questionnaire was considered as informed consent.

### Data analysis

The data were analysed statistically using SPSS 22.0 software (SPSS Inc., Chicago, Illinois). First, descriptive statistics (frequencies, percentages, means and standard deviations) were calculated to describe foot health and background factors. Second, the sum variables of overall foot health, skin, nails, structure and pain were formulated by counting the item scores and dividing the sum by the number of items. Third, the associations between foot health sum variables and background factors were tested using one-way analysis of variance and logistic regression analysis. In pairwise comparisons, Sidak adjustments for multiple comparisons were conducted. The statistical significance level was set to 0.05. The reliability of the S-FHAI was analysed using the Kuder–Richardsson Formula 20 coefficient.

## Results

### Survey response

The paper-based survey and a pre-paid return envelope were sent to all members fulfilling the inclusion criteria (*n* = 1318). In total, 504 surveys were returned, giving an overall response rate of 38%. Five surveys were excluded from the study because of empty responses and four because of being under the specified age range, resulting in 495 complete usable returned surveys.

### Description of participants

The majority of participants were female (*n* = 414, 84%). The respondents’ mean age was 64.95 years (range 21–88, SD 12.6). The most common diagnosed rheumatic conditions were rheumatoid arthritis (51%), osteoarthritis (26%), fibromyalgia (25%), ankylosing spondylitis (13%) and osteoporosis (10%). Some of the participants were diagnosed with more than one rheumatic condition. The respondents’ highest educational attainment level was elementary school (*n* = 157, 32%), primary school (*n* = 180, 36%) and high school or above (*n* = 154, 31%).

The mean of the self-evaluated score for foot health was 6.5 (range 0–10, SD 1.99, Table 1). In general, the participants considered their foot health to be very important (*n* = 339, 69%) or important (*n* = 135, 27%). Many participants reported walking either a lot (*n* = 143, 29%) or a little (*n* = 82, 17%) during the day. They also reported that their foot health affected their performance of their daily activities (*n* = 116, 23%). A little over half of the respondents had sought medical or nursing care for their foot problems (*n* = 261, 53%). One in ten (*n* = 59, 12%) reported being on sickness absence from their work because of foot problems.

**Table 1:** Background characteristics of the respondents (*n* = 495)

Background variable	*n* (%)
Mean age (years)	64.95 (range 21–88)
Gender	
Male	79 (16)
Female	414 (84)
Basic education	
Elementary school	157 (32)
Primary school	180 (37)
High school	154 (31)
Professional education	
Short vocational education	85 (17)
School-level vocational training	128 (26)
Polytechnic degree	141 (29)
University degree	79 (16)
No professional education	50 (10)
Occupation	
Manager	16 (3)
Employee	117 (24)
Self-employed	22 (4)
Student	3 (1)
Pensioner	330 (67)
Unemployed	6 (1)
Diagnosed rheumatic condition^a^	
Rheumatoid arthritis	251 (51)
Osteoarthritis	130 (26)
Fibromyalgia	124 (25)
Ankylosing spondylitis	63 (13)
Osteoporosis	49 (10)
Sjögren’s syndrome	30 (6)
Systemic lupus erythematosus	22 (4)
Hypermobility of joints	22 (4)
Polymyalgia rheumatica	17 (3)
Psoriatic arthritis	15 (3)
Juvenile idiopathic arthritis	13 (3)
Gout	12 (2)
Other rheumatic conditions¤	42 (8)
Self-perceived importance of foot health	
Very important	339 (69)
Important	135 (27)
Somewhat important	19 (4)
Not very important	2 (0.4)
Amount of daily walking or standing	
Very much	33 (7)
A lot	143 (29%)
Much or less	216 (44)
A little	82 (17)
Very little	20 (4)
Medical or nursing care because of foot problems	
Yes	261 (53)
No	233 (47)
Effect of foot health on daily activities	
Very much	102 (21)
Much	170 (36)
Much or less	116 (23)
A little	64 (13)
Very little	23 (7)
Sickness leave from work because of foot problems	
Yes	60 (13)
No	404 (87)
Self-evaluated level of foot health	
0	4 (1)
1	4 (1)
2	8 (2)
3	24 (5)
4	42 (9)
5	60 (12)
6	67 (14)
7	103 (21)
8	109 (22)
9	59 (12)
10	11 (2)

Lyme disease, mixed connective tissue disease, reactive arthritis, polymyositis, spondyloarthropathy, systemic sclerosis, vasculitis

^a^Respondents were able to select multiple responses

### The point prevalence of foot problems in a cohort of people living with a rheumatic condition

The number of respondents self-reporting the presence of a foot health complaint was 489. Thus, the point prevalence was 99 (489/495).

### Range and frequency of foot problems reported by people living with a rheumatic condition

The respondents reported a wide variety of foot problems (Table 2). Overall the S-FHAI mean score was 29 (SD 3.1, range 22–39), indicating the degree of the overall score for foot problems.

**Table 2:** Self-reported foot health problems in patients with rheumatic conditions (*n* = 495)

Abbreviated item	*f*	%
Foot skin		
Skin breaks or maceration between toes	68	14
Dry skin	335	68
Fissures in the heel	149	30
Corns or calluses	248	50
Verrucae	35	7
Blisters	17	3
Oedema	246	50
Sweating feet	110	22
Burning feet	113	23
Cold feet	280	57
Leg cramps	218	44
Foot ulcers	17	3
Toenails		
Ingrown toenail	101	20
Thickened nail	285	58
Colour changes in the nails	164	33
Fungal infection of the nails	51	10
Foot structure		
Hallux valgus	204	41
Taylor’s bunion	122	25
Hammer toe	214	43
Low foot arch	180	37
High foot arch	69	14
Foot pain	362	73

Concerning skin health, most respondents noted dry skin (*n* = 335, 68%) and cold feet (*n* = 280, 57%). Half of the respondents reported having corns or calluses (*n* = 248, 50%) and foot oedema (*n* = 246, 50%). Approximately one-third (*n* = 218, 44%) had leg cramps and fissures in the heel (*n* = 149, 30%). One-fifth reported foot sweating (*n* = 110, 22%) or burning feet (*n* = 113, 23%). The most seldom reported foot skin problems were skin breaks or maceration between the toes (14%), verrucae (7%), blisters (3%) and foot ulcers (3%).

Regarding toenail health, the most common problem was thickened toenails (*n* = 285, 58%). About one-third (33%) reported colour changes in the nails, and one-fifth (20%) ingrowing toenails. A minority of participants (*n* = 51, 10%) reported having a fungal infection in the nails. Regarding foot structure, hammer toe (*n* = 214, 43%) and hallux valgus (*n* = 204, 41%) were reported as the most prevalent structural problems. One-third of the participants had low foot arch (*n* = 180, 35%), one-quarter reported having Taylor’s bunion, and a few had high foot arch (*n* = 69, 14%).

**Fig. 1:**
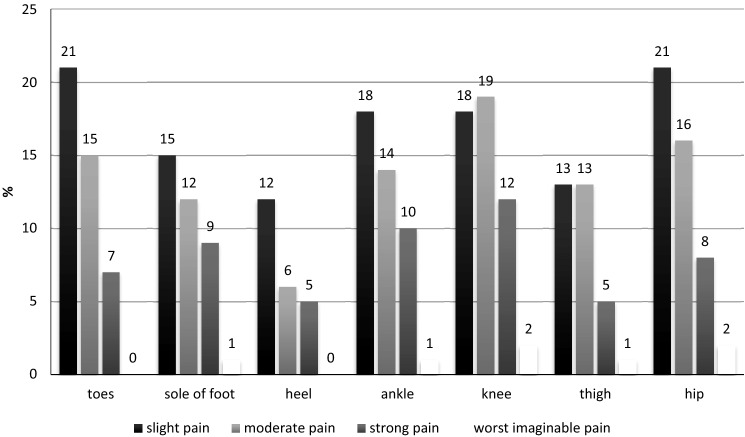
Self-reported foot and leg pain and pain location among people with rheumatic conditions (*n* = 495)

Most participants (*n* = 362, 73%) reported foot and leg pain (Fig. 1). Slight pain was most commonly reported in the area of the toes (21%) and hip (21%). Moderate pain was located in the knee (19%) and thigh (13%), and strong pain in the knee (12%) and ankle (10%). The worst imaginable pain was experienced in the sole of the foot (1%), ankle (1%), knee (2%), thigh (1%) and hip (2%) by a few respondents.

### Background factors associated with foot health in patients with rheumatic conditions

Some associations between reported foot health problems and the background factors of the participants were found. Participants with a high school education had worse foot health in general than those with elementary school-level education (*p* = 0.018). Foot health was worse in participants who stood very much every day compared to those who stood very little (*p* = 0.010). Participants who had to seek medical or nursing care because of foot problems had more foot problems (*p* = 0.015). Participants who wore walking or athletics shoes had better foot health (*p* = 0.009, *p* = 0.001, respectively).

Some associations with participants’ rheumatic disease type were identified. Participants with OA had poorer skin health (*p* = 0.011), more foot pain (*p *= 0.013) and in total more foot problems (*p* = 0.010) than participants with RA. However, no significant associations were found regarding which background factors are associated with the abovementioned problems. Foot health among participants with RA had some associations with background factors. Male participants with RA had poorer skin (*p* = 0.032) and nail health (*p* = 0.034) but fewer foot structural problems (*p* = 0.010) compared to patients with OA. However, the low sample size underpinning this calculation should be considered.

Participants older than 66 years had 1.497 higher odds (ß 1.497, 95% CI range 1.015–2.208, *p* = 0.042) of having more foot problems. Similarly, participants with arthritis had 1.783 (ß1.782, 95% CI range 1.176–2.704, *p* = 0.006) higher odds of having more foot problems.

## Discussion

Our data suggest a high proportion of foot health concerns across a broad cohort of people living with rheumatic and musculoskeletal long-term conditions. The most commonly reported problems were foot pain, dry skin, thickened toenails and cold feet. Poor foot health was statistically significantly associated with educational background (*p* = 0.018), amount of daily standing on feet (*p* = 0.010) and seeking medical or nursing care due to foot problems (*p* = 0.015).

The foot problems identified in this study were similar to those previously reported [30]. However, the frequency of foot pain was high in this sample, with foot and leg pain reported by the majority (73%) of respondents. Foot pain, if prolonged, can restrict people’s functional ability and may lead to an increased risk of falling, particularly in the older population [28]. It is unclear to what extent our data accurately reflect the true prevalence of foot pain relative to a general population due to potential sampling or reporting bias; however, arguably, the data does highlight a considerable potential unmet need with potential for further health related deterioration.

More than half of the respondents with rheumatic conditions had either dry skin or corns or calluses on their feet. Dry skin is a common foot problem in general [29], but also among people with rheumatic conditions [15]. Corns and calluses form easily on dry skin, where the elasticity and moisture in skin cells are decreased [30]. Untreated corns or calluses could become painful, thus limiting walking and causing changes in the foot's function, leading to additional disability in some other parts of the lower extremities. Furthermore, there is limited evidence to date, concerning the natural history of pedal skin health in people with rheumatic and musculoskeletal conditions, in particular routes to altered mechanical function, or ulceration and amputation [31]. Thus further research in this area is recommended and it may be beneficial to explore the efficacy of self-management or other personalised approaches to support pedal skin care [32]. The dry skin, corns and calluses may be considered minor foot problems.

Arguably, Hallux valgus, hammer toes and corns and calluses on the sole of the foot are clinical signs of altered function of foot biomechanics [31]. It is unclear from our survey data to what extent this is true, and further research exploring the potential association between local and systemic clinical signs, symptoms, and gross kinematic or kinetic function is potentially warranted given the high prevalence of signs indicated in this study [3]. Thus, Podiatry/ foot health services may play an important role in the diagnosis and monitoring phases of rheumatic disease [33].

Half of the respondents had visited a health care professional because of foot problems. Previous evidence suggests that people with rheumatic and musculoskeletal disease frequently consider their feet to be neglected in the context of broader medical consultations [34, 35]. The findings of this study appear to substantiate this, with a high proportion of respondents seeking additional support outside of specialist centre consultations. Provision of professional foot care is arguably necessary to promote foot health and support people coping with their changing foot health status. As such, easy access to foot care is needed to ensure the timely identification of problems and appropriate personalised care or referral for further consultation and further work to develop evidence-informed, internationally standardised, clinical guidance and support for workforce development is needed [3, 23].

### Limitations

The study has several limitations, particularly concerning the pragmatic approach to cohort data collection and limited inferential statistical analyses that can be applied. The data were collected from one regional area in southwest Finland, potentially limiting the generalisability of the study findings.

A self-administered Foot Health Assessment Instrument was used to collect the data. Diagnoses were determined as self-reported and participants were able to respond to more than one option in diagnosis. This may have caused a diagnostic confusion as diagnoses were not confirmed from patient records, as is the nature of self-reported survey. Thus, it was not possible to conduct a test of diagnostic accuracy or evaluate potential responder bias within the observed cohort. Based upon previously published literature concerning survey response bias, we anticipate that it is likely that the survey may over-represent the true rate of cases within a similar population. However, given the high prevalence observed, we suggest that that even a lower estimate would still represent a clinically significant prevalence rate, that would suggest further study is of clinical value. We also note that the overall response rate was moderate (38%) and in keeping with similar studies of this kind.

The S-FHAI is a self-assessment instrument, thus posing some threats to internal validity, as participants may assess their foot health problems differently despite the detailed assessment criteria in the S-FHAI. The internal consistency of the S-FHAI was satisfactory (0.720 with the Kuder–Richardsson Formula).

A pragmatic approach to the sampling frame, (survey geographical distribution, sample size, etc.) was used to enable evaluation of readily collected health data, rather than collecting new additional data within a sampling frame determined a priori. Thus, the data is subject to several omissions that may have been helpful in expanding upon demographical context, (the persons’ situation), or accounting for exposure variables known to also impact foot health, such as Body Mass Index (BMI). Therefore, we have interpreted our results with modesty and limited any inferential statistical analyses. None the less, the data do appear to suggest that further, more robust epidemiological study, is warranted.

Standardisation or adjustment within the analyses (e.g., for age) was not completed for the data analysis. A formal sample size calculation was not conducted based on known disease/foot problem estimates. However, as noted above, a pragmatic approach was applied. Nonetheless, the point prevalence estimate determined in this study can be used to help to inform future epidemiological studies of mixed rheumatic condition cohorts.

The cross-sectional design restricts the analysis of causal relationships. Therefore, future studies with longitudinal research designs are needed. There is possible unknown confounding within association analyses, and further longitudinal observational analysis is needed to explore potential relative exposure risks in more detail. However, this work highlights possible novel exposures (e.g., educational attainment) that have as yet been unidentified.

## Conclusions

People with rheumatic conditions reported many foot problems. The point prevalence of foot problems was high, particularly pain, thus suggesting a potential unmet need in people living with any rheumatic condition. Perceived foot problems were associated with peoples’ educational attainment, amount of daily standing on feet, and seeking of medical or nursing care because of foot problems. The study identified potential new bio-psycho-social variables that may be contributing to foot health that warrant future study. Future care guidelines accounting for biopsychosocial approaches to personalised foot care could advance people’s readiness, knowledge and skill to care for their own feet; however, further research is needed to clarify and substantiate this.

## Data Availability

The data sets used and/or analysed during the current study are available from the corresponding author upon reasonable request.
